# Sex differences in symptom network structure of depression, anxiety, and self-efficacy among people with diabetes: a network analysis

**DOI:** 10.3389/fpubh.2024.1368752

**Published:** 2024-03-01

**Authors:** Daoai Wu, Zhaoming Shi, Chenchen Wu, Weihua Sun, Guoxi Jin

**Affiliations:** Department of Endocrinology and Metabolism, The First Affiliated Hospital of Bengbu Medical University, Bengbu, China

**Keywords:** diabetes, depression, anxiety, self-efficacy, sex difference, network analysis

## Abstract

**Aims:**

The present study aims to explore the relations between symptoms of depression and anxiety and self-efficacy among people with diabetes. At the same time, we also examined the sex difference between network structures.

**Methods:**

This study recruited 413 participants with diabetes, and they completed Generalized Anxiety Disorder Scale (GAD-7), Patient Health Questionnaire (PHQ-9), and the Self-efficacy for Diabetes (SED). Symptom network analysis and network comparison test were used to construct and compare the depression-anxiety symptom network models of the female and male groups. Finally, we conducted flow diagrams to explore the symptoms directly or indirectly related to self-efficacy.

**Results:**

The strongest edges in the depression-anxiety symptom networks are the edge between “GAD3” (Excessive worry) and “GAD4” (Trouble relaxing) and the edge between “PHQ1” (Anhedonia) and “PHQ4” (Energy) in the female and male groups, respectively. Most of the symptoms with the highest EI and bridge EI are related to worry and nervousness. Additionally, in the flow diagram of the female group, “PHQ6” (Guilt) has a high negative association with self-efficacy.

**Conclusion:**

Females with diabetes are more vulnerable to depression and anxiety. Interventions targeting key symptoms in the network may be helpful in relieving the psychological problems among people with diabetes.

## 1 Introduction

Diabetes is a group of serious, long-term, and metabolic disorders that will have a major impact on not only individuals’ physical health but also their mental health (1, 2). Previous research suggests that individuals with diabetes are twice as likely to suffer from depression and anxiety as the general population (3, 4). Developing depression or anxiety will affect an individual’s job performance, quality of life, and even cause suicidal ideation (5–7). Additionally, suffering from mental health problems may also have a negative association with the self-efficacy of the diabetes population (8). Lower self-efficacy for diabetes may affect self-management behaviours in diabetic patients, exposing diabetic patients to the risk of diabetes-related morbidity and mortality (9). In other words, mental health problems may also affect the way individuals cope with diabetes, forming a negative circle. Things will get worse if individuals with diabetes have a comorbidity of depression and anxiety (10, 11).

Although a wealth of research about individuals with diabetes focuses on the relations between depression, anxiety, and self-efficacy, the symptom-level and detailed knowledge are relatively unknown. Considering the limitations of previous literature, the current study employed symptom network analysis to explore the symptom-level relation between depression, anxiety, and self-efficacy among people with diabetes. The present research can provide us with information on key symptoms that can be targeted when intervening in the mental health problems of people with diabetes and improving self-efficacy.

### 1.1 Depression and anxiety among individuals with diabetes

Diabetes is a kind of metabolic disorder characterised by high blood glucose levels (1). The results of the International Diabetes Federation (IDF) show that the global diabetes prevalence among people aged 20–79 years old in 2021 is about 10.5%, and this prevalence may rise to 12.2% in 2045 (12). The high prevalence and severe negative outcomes (i.e., financial burden, disability, and mortality) of diabetes make it an illness of worldwide concern (13, 14). China also has a large diabetes population, roughly 24% of the world’s diabetes population (13, 15). A large sampled cross-sectional study showed that the estimated overall prevalence of diabetes in mainland China in 2018 was 12.4% (16). These data highlight the significance of paying attention to the individuals with diabetes in China.

The mental health of individuals with diabetes deserves attention. The long-term dietary restrictions required to manage glycemic levels and the impact of diabetes on social and family functioning can contribute to mental health issues like depression and anxiety (4, 17). A burgeoning body of literature has identified the association between diabetes, depression, and anxiety (7, 18). The analysis of Li et al. (19) showed that the prevalences of depressive and anxious symptoms among individuals with type 2 diabetes in China were 37.8 and 28.9%, respectively, which were higher than the prevalences in the general population. Mental health problems such as depression and anxiety have negative effects on people’s quality of life and are even associated with suicidal ideation (6, 7). What’s more, it is documented that mental health problems are also related to reductions in self-efficacy for diabetes, which may have an impact on the physical health of the diabetes population (8, 9). Individuals who suffer from a co-morbidity of anxiety and depression may face more serious negative consequences (10, 11). In sum, considering the high prevalence and the detrimental outcomes of depression and anxiety in individuals with diabetes, the issues of mental health problems in the diabetes population warrant attention.

### 1.2 Depression, anxiety, and self-efficacy for diabetes

Effective diabetes treatment needs changes in the patient’s daily routine (i.e., insulin injections, blood glucose testing, and diet), which means that the self-management of individuals plays a significant role (20). Self-efficacy, the perception of one’s capability to overcome difficulties and perform specific behaviours to achieve his or her goals (20), is a key factor that affects the self-management behaviours of diabetes individuals (21). Jiang et al. (22) employed a meta-analysis containing 1,308 participants, finding that enhancing self-efficacy-focused education on diabetes would enhance self-management behaviours and improve the quality of life. Consistently, other studies also supported the high association between self-efficacy and self-management behaviours among people with diabetes (21, 23), highlighting the significance of improving self-efficacy of the diabetes population.

Research showed that negative mood states would have a negative effect on self-efficacy (24). It was identified that depression and anxiety share a negative association with self-efficacy in people with diabetes (8, 25, 26). Additionally, the relationship between depression, anxiety and self-efficacy may differ in males and females. The prevalences of depression and anxiety among the diabetes population (27) and the level of self-efficacy (28) are related to sex. The research of Cherrington et al. (25) showed that negative associations between depression and self-efficacy existed in males with diabetes but not females with diabetes. Shakeel et al. (29) found that the relation between anxiety and self-efficacy of patients with chronic illness varied across genders. Thus, it is necessary to consider sex when analyzing the relationship between depression, anxiety, and self-efficacy among the diabetes population.

### 1.3 The current study

Reviewing previous literature, several gaps need to be closed. Firstly, even though previous research has identified the relationship between mental health problems (i.e., depression or anxiety) and diabetes, few of them take the perspective of network analysis to examine the symptom-level relation between the comorbidity of depression and anxiety among individuals with diabetes. Secondly, although studies have found that psychological problems are associated with lower self-efficacy, the evidence about which symptoms of anxiety and depression are more strongly associated with self-efficacy for diabetes is limited. Thirdly, current knowledge of sex differences in the relationship between depression, anxiety, and self-efficacy is limited to the symptom levels, which warrant further exploration.

To address the aforementioned deficits, symptom network analysis, a popular method in the clinical psychology area to provide detailed information on symptom-level relations between different variables (30), is suitable. The current study applied network analysis to examine the symptom-level relation between depression, anxiety, and self-efficacy, considering the sex difference. There are three aims in the present study. First of all, we aim to construct the depression-anxiety symptom network models of the diabetes population, identifying the key symptoms in the network structures. Second, we constructed the flow diagrams including self-efficacy and symptoms of depression and anxiety. Through this procedure, we can find the important symptoms that are related to self-efficacy. Third, we aim to examine whether sex differences existed in the depression-anxiety symptom network models and the flow network models.

## 2 Method

### 2.1 Measures

#### 2.1.1 Generalized anxiety disorder scale

The Generalized Anxiety Disorder Scale has 7 items, which is used to measure the level of anxiety of individuals (31). Each item scored on a 4-point Likert scale, ranging from 0 (not at all) to 3 (nearly every day). The total score of this scale ranges from 0 to 21 and a higher score of GAD-7 represents more severe anxious symptoms. Previous studies show the applicability of its Chinese version (32). With a Cronbach’s α score of 0.943, GAD-7 shows great internal consistency in the present study.

#### 2.1.2 Patient health questionnaire

The 9-item Patient Health Questionnaire is applied to measure the severity of depressive symptoms (33). Each of the items is rated from 0 (not at all) to 3 (nearly every day) and the higher total scores of this questionnaire indicate the more severe depressive symptoms. The Chinese version of PHQ-9 shows good psychometric properties (34). In the current study, the Cronbach’s α score of PHQ-9 is 0.884.

#### 2.1.3 The self-efficacy for diabetes

The current study applied the Chinese version of SED to measure the level of self-efficacy in people with diabetes to cope with diabetes (35). This scale has 9 items and each scored from 1 (no confidence at all) to 5 (with complete confidence), with higher total scores indicating a higher level of self-efficacy. The Cronbach’s α score of SED is 0.890.

### 2.2 Participants and procedure

This survey used a convenience sampling method to collect the data, which was conducted in June 2023 in Bengbu, Anhui Province, China. For the fact that most of the subjects participating in the current study are the older and less educated individuals, it was hard for them to complete the questionnaire independently. Therefore, in this study, after obtaining informed consent from the participants, the nursing staff read the questions to the subjects, asked them for their answers and recorded their answers on a paper questionnaire. After completing the questionnaire, one insulin was given to each participant as payment. Finally, the staff converted these paper-based questionnaires into an electronic version data. This study recruited 413 individuals with diabetes (*Mean _age_* = 50.68, *SD _age_* = 13.31) to complete the questionnaire. The average duration of their diabetes of them is 7.40 years. To further examine the symptom network differences between males and females, we divided them into two different groups according to their sexes. The female group has 147 individuals (*Mean _age_* = 50.32, *SD _age_* = 14.54) and the male group has 266 individuals (*Mean _age_* = 50.88, *SD _age_* = 12.61). The average years of diabetes in female group and male group are 7.94 and 7.10, respectively. This research was examined and approved by the ethics committee of Bengbu Medical University (Reference number: No. 148 [2021]).

### 2.3 Data analysis

The current study used R (version 4.3.1) to analyse the data (36). In the beginning, we conducted a descriptive analysis to describe the basic information of the female group and the male group. Additionally, using the function *descrTable* of R package *compareGroups* (37), we also conducted a *t*-test to compare the age and scores of depression, anxiety, and self-efficacy between the female group and the male group. Secondly, to know the detection rates of anxiety and depression among the individuals with diabetes in the current sample, we calculated the prevalences of depression and anxiety according to the cut-off scores. Specifically, the cut-off scores of PHQ-9 and GAD-7 are 8 (33) and 7 (38), respectively.

To estimate the relations between different symptoms and construct the network model, the Gaussian Graphical Model (GGM) was conducted (39). However, to avoid the network being too complex to be understood, the GGM needed to be further regularized through the Extended Bayesian information criterion (EBIC) and graphical least absolute shrinkage and selection operator (LASSO) (40, 41). Then, we used the R package *qgraph* to achieve the visualization of the symptom network (42). In the present study, we constructed depression-anxiety symptom network models of males and females to examine the relations between symptoms of depression and anxiety. In the symptom network, each node represents a symptom of depression or anxiety. The edge represents the association between two symptoms and a thicker edge means a stronger association. Additionally, the green line and red line represent positive and negative relationships, respectively.

After constructing the symptom network models, we calculated the centrality indexes of each node to identify the significant symptoms of network models. First of all, we computed the Expected Influence (EI) of each symptom through the R package *qgraph* (42). This index is the sum of all positive and negative edge weights connected to a specific node, which is a reliable index to measure the significance of each node in the network model (43). Then, the R package *mgm* was used to calculate the predictability (i.e., *R*^2^) of each node (44). *R*^2^ measures the variance that a node can be explained by other neighbouring nodes in the network structure (45). Third, for the reason that the network models in the present study include depression and anxiety two disorders, to identify the important nodes that connected two disorders, we computed the bridge expected influence (bridge EI) of each node. Bridge EI is calculated in a similar way as EI. It is the sum of a node’s edge weights, but only edges that connect nodes from one disorder with the other disorder are counted (46). Referring to previous studies, the symptoms with bridge EI higher than 1 were identified as bridge symptoms in the network (47).

To examine the accuracy and stability of the network models, we applied the R package *bootnet* (40). To test the accuracy, we evaluate the bootstrapped confidence intervals (95% CIs) by using a nonparametric bootstrap. A narrower CI means a more reliable network model. Additionally, to test if there is a significant difference between the edge weights or the centrality indexes (i.e., EI and bridge EI) of two symptoms, we also conducted a bootstrapped difference test. To examine the stability of the network model, we computed the correlation stability – coefficients (CS-C) of EI. The CS-C represents the maximum proportion of the sample size that can be excluded while maintaining a correlation coefficient between the centrality index of the original sample and the after-dropped sample at least 0.7, with 95% probability. According to the criterion of previous research, the CS-C should be higher than 0.25 and it is preferable to be higher than 0.5 (48).

We also performed a Network Comparison Test (NCT) through the R package *NetworkComparisonTest* to evaluate the difference in the depression-anxiety network models between females and males (49). Using NCT, we can compare whether there are differences in global connectivity and local connectivity between two groups.

Aiming at identifying the significant symptoms of depression and anxiety that are directly connected with the self-efficacy of diabetes, we employed the function *flow* to construct the flow diagram containing self-efficacy of diabetes and symptoms of depression and anxiety. The flow diagram places self-efficacy of diabetes on the left side and shows us how symptoms of depression and anxiety are directly or indirectly connected to self-efficacy of diabetes.

## 3 Results

The descriptive information of the current sample and the *t*-test results between the female group and the male group are shown in Table 1. Specifically, according to the results of Table 1, we can find that the total scores of depression and anxiety have significant differences between two groups. The female group has higher total scores of depression and anxiety. The prevalences of depression and anxiety in the present sample are 43.82 and 31.72%. The prevalences of depression and anxiety in the female group are 52.38 and 38.10%. In the male group, the prevalences of depression and anxiety are 39.10 and 28.20%.

**Table 1:** The descriptive information of female group and male group and the results of *t*-test between two groups.

Variables	Labels	Female group (*n* = 147)	Male group (*n* = 266)	*p*
		Mean (SD)	Mean (SD)	
age		50.32 (14.54)	50.88 (12.61)	0.697
PHQ1	Anhedonia	1.11 (0.94)	1.06 (0.90)	0.610
PHQ2	Sad Mood	0.97 (0.94)	0.71 (0.84)	0.005
PHQ3	Sleep	1.28 (0.98)	1.05 (0.98)	0.025
PHQ4	Energy	1.29 (1.03)	1.21 (0.92)	0.485
PHQ5	Appetite	1.16 (0.95)	1.00 (0.90)	0.083
PHQ6	Guilt	0.79 (0.99)	0.66 (0.90)	0.183
PHQ7	Concentration	0.78 (0.96)	0.68 (0.95)	0.302
PHQ8	Motor	0.66 (0.84)	0.55 (0.81)	0.195
PHQ9	Suicidal ideation	0.43 (0.73)	0.25 (0.62)	0.014
GAD1	Nervousness	0.85 (0.89)	0.76 (0.88)	0.298
GAD2	Uncontrollable worry	0.82 (0.99)	0.60 (0.82)	0.020
GAD3	Excessive worry	1.02 (1.05)	0.78 (0.89)	0.019
GAD4	Trouble relaxing	0.88 (1.00)	0.68 (0.89)	0.040
GAD5	Restlessness	0.56 (0.84)	0.54 (0.86)	0.788
GAD6	Irritability	0.97 (0.96)	0.86 (0.90)	0.260
GAD7	Feeling afraid	0.73 (0.97)	0.50 (0.81)	0.012
Depression		8.47 (5.96)	7.17 (5.71)	0.033
Anxiety		5.84 (5.90)	4.71 (5.14)	0.051
SED		27.35 (7.19)	27.95 (7.68)	0.434

### 3.1 Network structure

**Figure 1:**
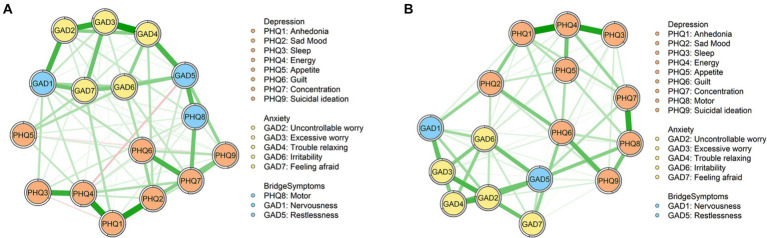
Network structures. **(A)** Depression-anxiety symptom network of the female group. **(B)** Depression-anxiety symptom network of the male group.

The depression-anxiety symptom network models of the female and male groups are depicted in Figure 1. Figure 1A is the network of the female group. This network has 16 nodes, with 75 non-zero edges (62.5%). Among these edges, the edges between “GAD3” (Excessive worry) and “GAD4” (Trouble relaxing), “PHQ1” (Anhedonia) and “PHQ2” (Sad Mood), and “PHQ1” (Anhedonia) and “PHQ4” (Energy) have the strongest correlation (refer to Supplementary Table S1) and they are significantly higher than other edges among the network (see Supplementary Figure S1A). Figure 1B shows the network of the male group. In this network, there are 16 nodes and 67 non-zero edges (55.83%). Among all non-zero edges, the top three strongest edges are the edges of “PHQ1” (Anhedonia) and “PHQ4” (Energy), “PHQ3” (Sleep) and “PHQ4” (Energy), and “PHQ7” (Concentration) and “PHQ8” (Motor) (see Supplementary Table S2), which are significantly higher than other edges in the network model (see Supplementary Figure S1B).

**Figure 2:**
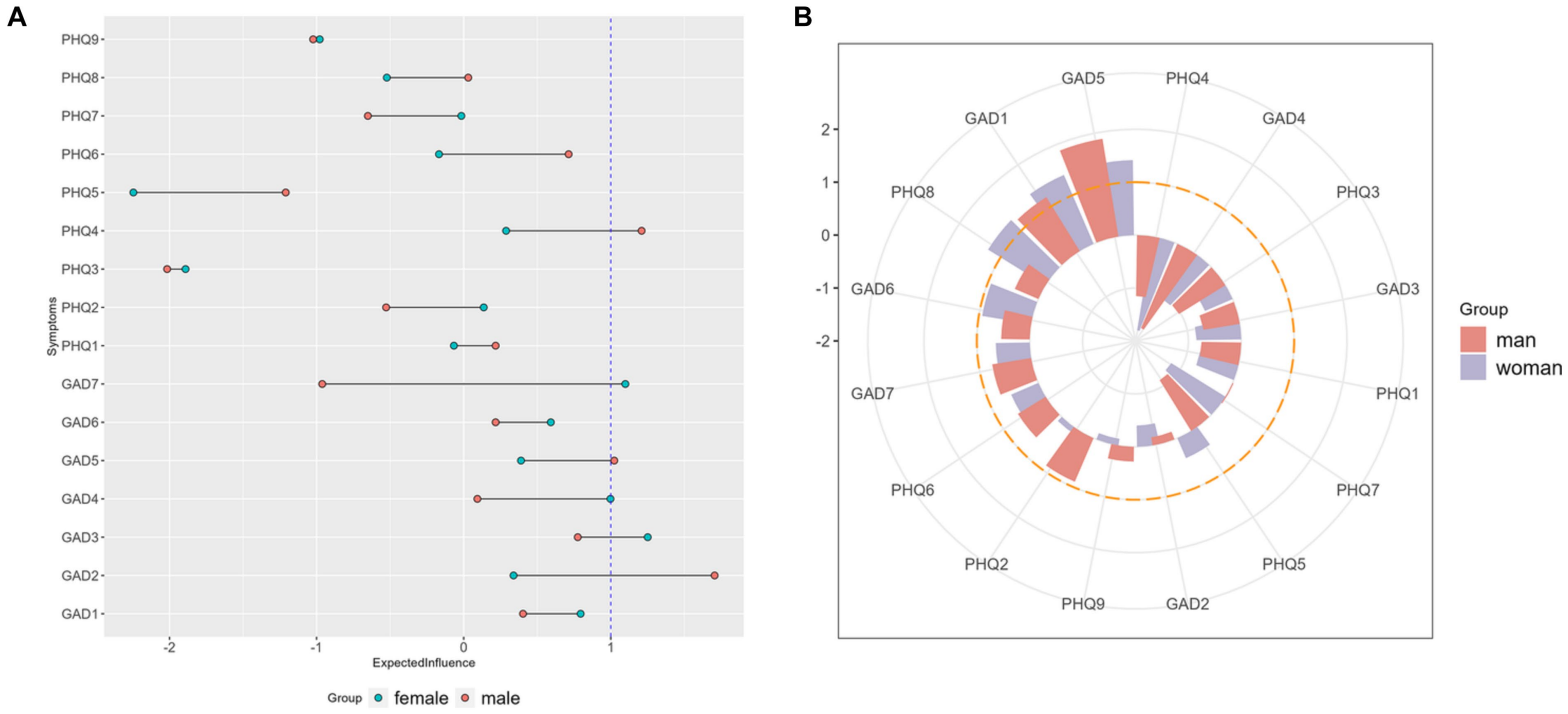
Standardized EI and bridge EI. **(A)** Standardized EI of each symptom in female and male groups. **(B)** Standardized bridge EI of each symptom in female and male groups.

Figure 2 shows the EI and bridge EI of the female group and the male group. Figure 2A is the EI of each symptom of the female group and male group. Among the female group, “GAD3” (Excessive worry), “GAD7” (Feeling afraid), and “GAD4” (Trouble relaxing) are the symptoms with the highest standardized EI. The EI of “GAD3” (Excessive worry) is significantly higher than 6 nodes in the network (see Supplementary Figure S2A). Differently, among the male group, the standardized EI of “GAD2” (Uncontrollable worry), “PHQ4” (Energy) and “GAD5” (Restlessness) ranked in the top three. The EI of “GAD2” (Uncontrollable worry) is significantly higher than the other 10 nodes in the male network (see Supplementary Figure S2B). The standardized bridge EI of symptoms is shown in Figure 2B. According to Figure 2B, “GAD1” (Nervousness) and “GAD5” (Restlessness) are the bridge symptoms of both the female group and male group, with standardized bridge EI higher than 1. Additionally, “PHQ8” (Motor) are the unique bridge symptom of the female group.

### 3.2 Network comparison

**Figure 3:**
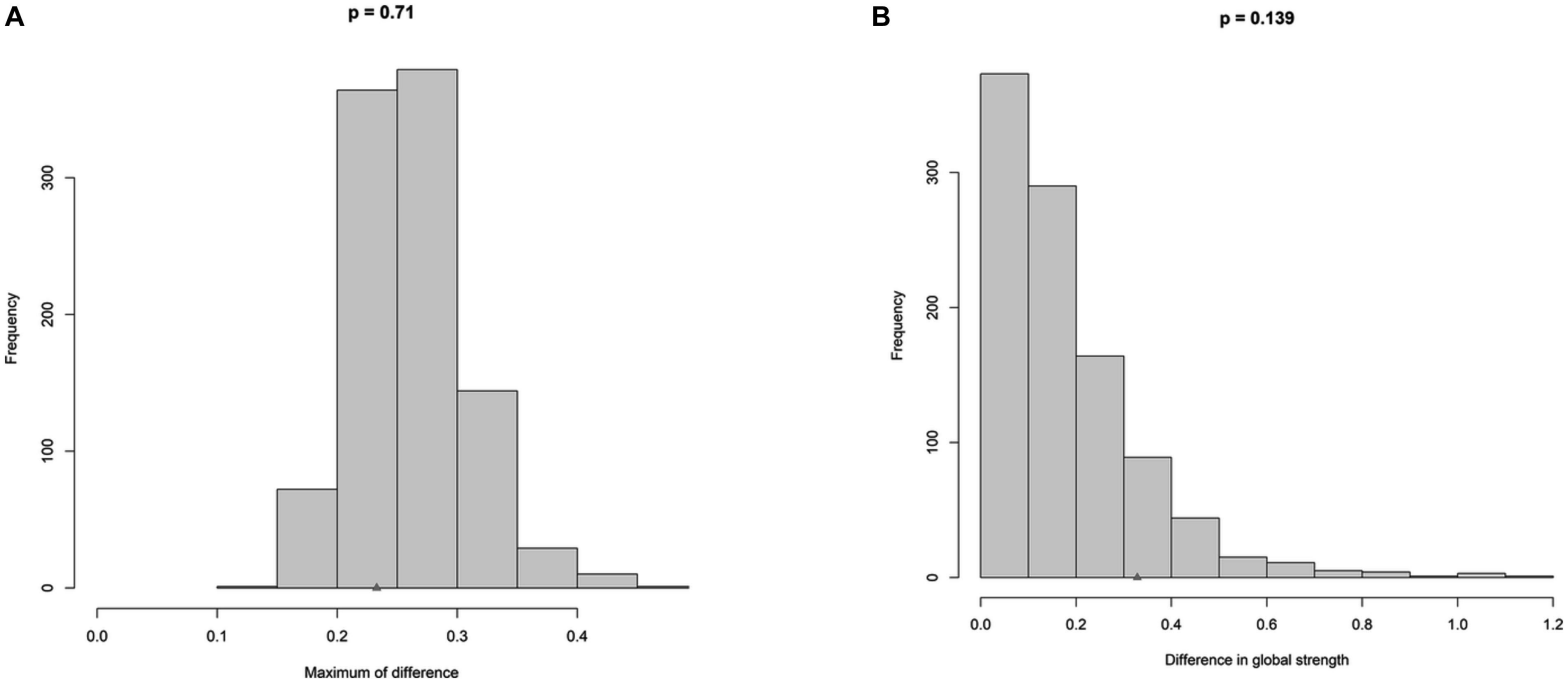
Comparison of network between trauma group and no-trauma group. **(A)** Differences in edge weights for the networks. **(B)** Differences in global strength for the networks.

Figure 3 depicts the results of network comparison between the female group and the male group. There are no significant differences in the distribution of edge weights (M = 0.233, *p* = 0.71) and global strength (S = 0.328, *p* = 0.139) between two groups.

### 3.3 Network stability and accuracy

The results of the bootstrapped analysis are depicted in Supplementary Figure S3. Additionally, Supplementary Figures S4, S5 show the results of case-dropping analysis. The CS-Cs of EI of the female group and male group are 0.592 and 0.594, respectively. In terms of the CS-Cs of bridge EI of two groups, the CS-C of the female group is 0.204 and the CS-C of the male group is 0.282.

### 3.4 Flow network

**Figure 4:**
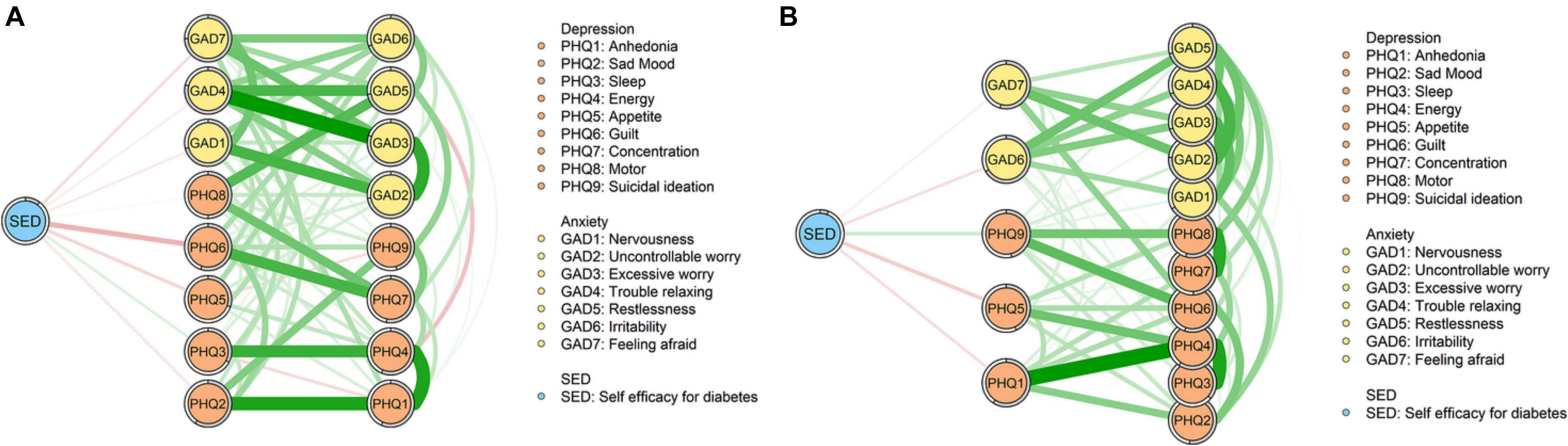
Flow network models. **(A)** Flow network of female group. **(B)** Flow network of male group.

The flow diagrams of the female group and the male group are shown in Figure 4. According to Figure 4, we can find that compared with the male group, the female group has more symptoms that are directly connected with self-efficacy. Among the symptoms that are directly linked with self-efficacy, “PHQ6” (Guilt) is the symptom with the strongest connection in the female group. In the male group, “PHQ5” (Appetite) is the symptom that has the strongest negative association with self-efficacy.

## 4 Discussion

The present study focuses on individuals with diabetes, examining the symptom-level relation between depression, anxiety, and self-efficacy. Several striking findings needed to be further explained.

According to the results of the *t*-test, we discovered that, compared to males, females have higher prevalences of depression and anxiety. This is consistent with the other research considering the sex difference, suggesting that, among people with diabetes, females have higher risks of developing depression and anxiety (27, 50). Additionally, similar to other studies, we also found that the mean level of self-efficacy of females with diabetes is lower (29). Previous research also identified that females seem to experience a greater impact of diabetes (51) and factors such as hormones, genes, and social roles may play a role (52). In other words, females facing diabetes may be more vulnerable to psychological problems, which need extra concern.

In terms of the results of depression-anxiety network, our analysis showed that the edge between “GAD3” (Excessive worry) and “GAD4” (Trouble relaxing) and the edge between “PHQ1” (Anhedonia) and “PHQ4” (Energy) are the strongest edges in the female and male group, respectively. The variation of the strongest edges may be related to the traditional social roles of different sexes. Traditionally, females usually need to be the caregiver and are expected to take care of household chores (53). This traditional perception of gender roles may be more typical in older individuals (54). Thus, females with diabetes in the current study may not only need to worry about their own physical and mental health but also need to take responsibility for other household chores and matters within the family. What’s more, the study also suggests that females are more anxious about the disease (55). These may contribute to the strongest edge between “GAD3” (Excessive worry) and “GAD4” (Trouble relaxing) among the female network. Differently, the traditional social role of males is that males usually take more outdoor and social activities. Additionally, males seem to have a higher level of sensation seeking than females (56). Pleasurable activities will inevitably be restricted due to diabetes, which may make males feel a lack of enjoyment. This may be the possible explanation for the strongest edge between “PHQ1” (Anhedonia) and “PHQ4” (Energy) in the male group.

With respect to the results of EI and bridge EI of each node, our study found that, in both the female group and male group, symptoms with the highest EI and two bridge symptoms are related to worry and nervousness. Stuckey et al. (57) explored the psychosocial experiences of diabetes and found two main negative psychosocial themes. One of them is the anxiety and fear about hypoglycemia and complications of diabetes. Apart from this, a review of literature about qualitative studies on the lived experience of individuals with diabetes also pointed out that individuals with diabetes have uncertainty about the future and are afraid of losing functions (58). These findings, together with the results of our analysis, suggest that the uncertainty and worry about illness and life among people with diabetes is a key point that needs to be solved.

Out of our expectation, the analysis of NCT did not yield significant results, which showed that the global strength between two groups did not have a significant difference. On one hand, this finding shows that the connectivity between the nodes of two groups did not have a difference. This indicates that the increased susceptibility to developing depression and anxiety among females with diabetes (as evidenced by higher mean levels and prevalence rates of depression and anxiety) is not solely due to a closer association between anxiety and depression symptoms, which are more likely to trigger and perpetuate each other (59). Instead, there may be other factors that put females with diabetes in a situation where they are more likely to have anxiety and depression. Factors like culture, gender roles, and genes may contribute to this (52, 60). On the other hand, the limitations of sample size may be associated with this not non-significant result. Thus, future studies with larger sample sizes are needed.

A striking difference existed in flow diagrams of female and male groups including symptoms of depression and anxiety, and self-efficacy, which shows the outstanding relation between “PHQ6” (Guilt) and self-efficacy in the female group but not male group. In the male group, “PHQ5” (Appetite) and self-efficacy have the strongest association. “PHQ6” (Guilt) represents the extent to which an individual feels bad about themselves and feels that they are letting their family down (61). Our finding is similar to the results of previous research, which observed that females with diabetes experienced more interpersonal distress and males with diabetes experienced more regimen-related distress (62). Additionally, research also documented that females with diabetes reported more weight stigma (63) and lower self-esteem (64) compared to males. Thus, we can infer that females with diabetes may suffer from more interpersonal distress and may have low self-esteem. This may have an association with self-efficacy (62), as the results in the present study. Differently, regimen-related distress, such as problems of appetite, has a high association with self-efficacy. According to this, the intervention targeting to relieve the feeling of guilt and self-blame may be helpful for females with diabetes to improve their self-efficacy. For males with diabetes, it may be necessary to increase their self-efficacy by reducing regimen-related distress.

## 5 Limitations

Although the current study shows fresh insights about the symptom-level relation between depression, anxiety, and self-efficacy among people with diabetes and examines the sex differences, several limitations need to be noted. First, the current study used self-report questionnaires and it was undoubtedly affected by subjectivity. Thus, future studies can try to use some objective indicators or combine the other-report questionnaire, which can provide more comprehensive and objective information. Second, most of the participants in this study were middle-aged individuals. Considering the psychological and physical characteristics of middle-aged individuals, the results derived from this study may not be generalisable to individuals of other ages. Third, the results of bootstrapped analysis and case-dropping analysis showed that the accuracy and stability are less satisfactory, which may be due to the limited sample of the study. Thus, future studies can further examine and validate the results of this study.

## 6 Conclusion

This research, recruiting participants with diabetes, aims to explore the symptom-level relations between depression, anxiety, and self-efficacy through network analysis. Additionally, the current study also examined whether there are sex differences. Our analysis showed that females with diabetes had higher prevalences of depression and anxiety, and scored lower in self-efficacy. As to the depression-anxiety symptom network of females, the edge between “GAD3” (Excessive worry) and “GAD4” (Trouble relaxing) is the strongest edge. In the male group, the edge between “PHQ1” (Anhedonia) and “PHQ4” (Energy) is the strongest. In terms of the key symptoms in the network models, symptoms with the highest EI and two bridge symptoms relate to worry and nervousness across two groups. Last, our research found that the relations between depression and anxiety symptoms and self-efficacy differ in female and male groups. “PHQ6” (Guilt) played a significant role in the flow network of females.

## Data availability statement

The original contributions presented in the study are included in the article/Supplementary material, further inquiries can be directed to the corresponding author.

## Ethics statement

The studies involving humans were approved by this research was examined and approved by the ethics committee of Bengbu Medical University (Reference number: No. 148 [2021]). The studies were conducted in accordance with the local legislation and institutional requirements. The participants provided their written informed consent to participate in this study.

## Author contributions

DW: Investigation, Methodology, Writing – original draft. ZS: Writing – review & editing. CW: Writing – review & editing. WS: Writing – review & editing. GJ: Conceptualization, Data curation, Funding acquisition, Writing – review & editing.
