# Supplementary material for: Sex differences in symptom network structure of depression, anxiety, and self-efficacy among people with diabetes: a network analysis
Source: Front Public Health. 2024 Mar 1;12:1368752. doi: 10.3389/fpubh.2024.1368752 (PMC10941846; doi:10.3389/fpubh.2024.1368752)
Supplement: Supplementary file 1 [file Data_Sheet_1.docx]

**Table S1** The matrix of female group

|  | PHQ1 | PHQ2 | PHQ3 | PHQ4 | PHQ5 | PHQ6 | PHQ7 | PHQ8 | PHQ9 | GAD1 | GAD2 | GAD3 | GAD4 | GAD5 | GAD6 | GAD7 |
| --- | --- | --- | --- | --- | --- | --- | --- | --- | --- | --- | --- | --- | --- | --- | --- | --- |
| PHQ1 | 0 | 0.307 | -0.048 | 0.305 | 0.050 | 0 | 0.082 | 0.046 | 0 | 0 | 0 | 0.019 | 0 | 0 | 0.020 | 0.106 |
| PHQ2 | 0.307 | 0 | 0.074 | 0.004 | 0 | 0.137 | 0 | 0 | 0.168 | 0.048 | 0 | 0 | 0.101 | 0.075 | 0 | 0.016 |
| PHQ3 | -0.048 | 0.074 | 0 | 0.268 | 0 | 0 | 0 | 0.069 | 0 | 0.093 | 0 | 0.048 | 0 | 0.026 | 0 | 0 |
| PHQ4 | 0.305 | 0.004 | 0.268 | 0 | 0.081 | 0.065 | 0.113 | 0.042 | 0.044 | 0.081 | 0 | 0.032 | 0 | -0.079 | 0 | 0 |
| PHQ5 | 0.050 | 0 | 0 | 0.081 | 0 | 0.056 | 0 | 0.047 | -0.044 | 0 | 0.054 | 0 | 0 | 0 | 0.113 | 0.106 |
| PHQ6 | 0 | 0.137 | 0 | 0.065 | 0.056 | 0 | 0.247 | -0.029 | 0.102 | 0.098 | 0.056 | 0.038 | 0 | -0.015 | 0.113 | 0 |
| PHQ7 | 0.082 | 0 | 0 | 0.113 | 0 | 0.247 | 0 | 0.194 | 0.168 | 0 | 0 | 0 | 0.013 | 0 | 0.085 | 0 |
| PHQ8 | 0.046 | 0 | 0.069 | 0.042 | 0.047 | -0.029 | 0.194 | 0 | 0.061 | 0.025 | 0.075 | 0 | 0 | 0.229 | 0 | 0.038 |
| PHQ9 | 0 | 0.168 | 0 | 0.044 | -0.044 | 0.102 | 0.168 | 0.061 | 0 | 0.038 | 0 | 0 | 0 | 0.146 | 0 | 0.031 |
| GAD1 | 0 | 0.048 | 0.093 | 0.081 | 0 | 0.098 | 0 | 0.025 | 0.038 | 0 | 0.249 | 0.080 | 0.027 | 0 | 0.131 | 0.195 |
| GAD2 | 0 | 0 | 0 | 0 | 0.054 | 0.056 | 0 | 0.075 | 0 | 0.249 | 0 | 0.281 | 0.161 | 0.027 | 0 | 0.062 |
| GAD3 | 0.019 | 0 | 0.048 | 0.032 | 0 | 0.038 | 0 | 0 | 0 | 0.080 | 0.281 | 0 | 0.343 | 0.044 | 0.072 | 0.198 |
| GAD4 | 0 | 0.101 | 0 | 0 | 0 | 0 | 0.013 | 0 | 0 | 0.027 | 0.161 | 0.343 | 0 | 0.243 | 0.148 | 0.064 |
| GAD5 | 0 | 0.075 | 0.026 | -0.079 | 0 | -0.015 | 0 | 0.229 | 0.146 | 0 | 0.027 | 0.044 | 0.243 | 0 | 0.160 | 0.122 |
| GAD6 | 0.020 | 0 | 0 | 0 | 0.113 | 0.113 | 0.085 | 0 | 0 | 0.131 | 0 | 0.072 | 0.148 | 0.160 | 0 | 0.178 |
| GAD7 | 0.106 | 0.016 | 0 | 0 | 0.106 | 0 | 0 | 0.038 | 0.031 | 0.195 | 0.062 | 0.198 | 0.064 | 0.122 | 0.178 | 0 |

**Table S2** The matrix of male group

|  | PHQ1 | PHQ2 | PHQ3 | PHQ4 | PHQ5 | PHQ6 | PHQ7 | PHQ8 | PHQ9 | GAD1 | GAD2 | GAD3 | GAD4 | GAD5 | GAD6 | GAD7 |
| --- | --- | --- | --- | --- | --- | --- | --- | --- | --- | --- | --- | --- | --- | --- | --- | --- |
| PHQ1 | 0 | 0.164 | 0 | 0.356 | 0.106 | 0.112 | 0.103 | 0.015 | 0 | 0.050 | 0 | 0 | 0 | 0 | 0.044 | 0 |
| PHQ2 | 0.164 | 0 | 0.032 | 0.041 | 0.091 | 0.182 | 0 | 0 | 0.077 | 0.149 | 0.094 | 0 | 0.003 | 0 | 0 | 0 |
| PHQ3 | 0 | 0.032 | 0 | 0.315 | 0.0003 | 0.060 | 0.061 | 0 | 0.059 | 0 | 0 | 0 | 0 | 0 | 0.037 | 0.031 |
| PHQ4 | 0.356 | 0.041 | 0.315 | 0 | 0.201 | 0 | 0.142 | 0 | 0 | 0 | 0 | 0 | 0 | 0 | 0.057 | 0 |
| PHQ5 | 0.106 | 0.091 | 0.0003 | 0.201 | 0 | 0.117 | 0.030 | 0.106 | 0.007 | 0 | 0 | 0 | 0 | 0 | 0.059 | 0 |
| PHQ6 | 0.112 | 0.182 | 0.060 | 0 | 0.117 | 0 | 0.014 | 0.099 | 0.225 | 0 | 0 | 0.049 | 0 | 0.017 | 0 | 0.154 |
| PHQ7 | 0.103 | 0 | 0.061 | 0.142 | 0.030 | 0.014 | 0 | 0.296 | 0 | 0 | 0 | 0.028 | 0 | 0.108 | 0.015 | 0.014 |
| PHQ8 | 0.015 | 0 | 0 | 0 | 0.106 | 0.099 | 0.296 | 0 | 0.194 | 0.041 | 0 | 0 | 0 | 0.167 | 0 | 0 |
| PHQ9 | 0 | 0.077 | 0.059 | 0 | 0.007 | 0.225 | 0 | 0.194 | 0 | 0.034 | 0.055 | 0.021 | 0 | 0.044 | 0 | 0.034 |
| GAD1 | 0.050 | 0.149 | 0 | 0 | 0 | 0 | 0 | 0.041 | 0.034 | 0 | 0.132 | 0.221 | 0.177 | 0.047 | 0.133 | 0 |
| GAD2 | 0 | 0.094 | 0 | 0 | 0 | 0 | 0 | 0 | 0.055 | 0.132 | 0 | 0.251 | 0.254 | 0.200 | 0 | 0.205 |
| GAD3 | 0 | 0 | 0 | 0 | 0 | 0.049 | 0.028 | 0 | 0.021 | 0.221 | 0.251 | 0 | 0.138 | 0 | 0.175 | 0.154 |
| GAD4 | 0 | 0.003 | 0 | 0 | 0 | 0 | 0 | 0 | 0 | 0.177 | 0.254 | 0.138 | 0 | 0.200 | 0.156 | 0 |
| GAD5 | 0 | 0 | 0 | 0 | 0 | 0.017 | 0.108 | 0.167 | 0.044 | 0.047 | 0.200 | 0 | 0.200 | 0 | 0.201 | 0.094 |
| GAD6 | 0.044 | 0 | 0.037 | 0.057 | 0.059 | 0 | 0.015 | 0 | 0 | 0.133 | 0 | 0.175 | 0.156 | 0.201 | 0 | 0.075 |
| GAD7 | 0 | 0 | 0.031 | 0 | 0 | 0.154 | 0.014 | 0 | 0.034 | 0 | 0.205 | 0.154 | 0 | 0.094 | 0.075 | 0 |

**
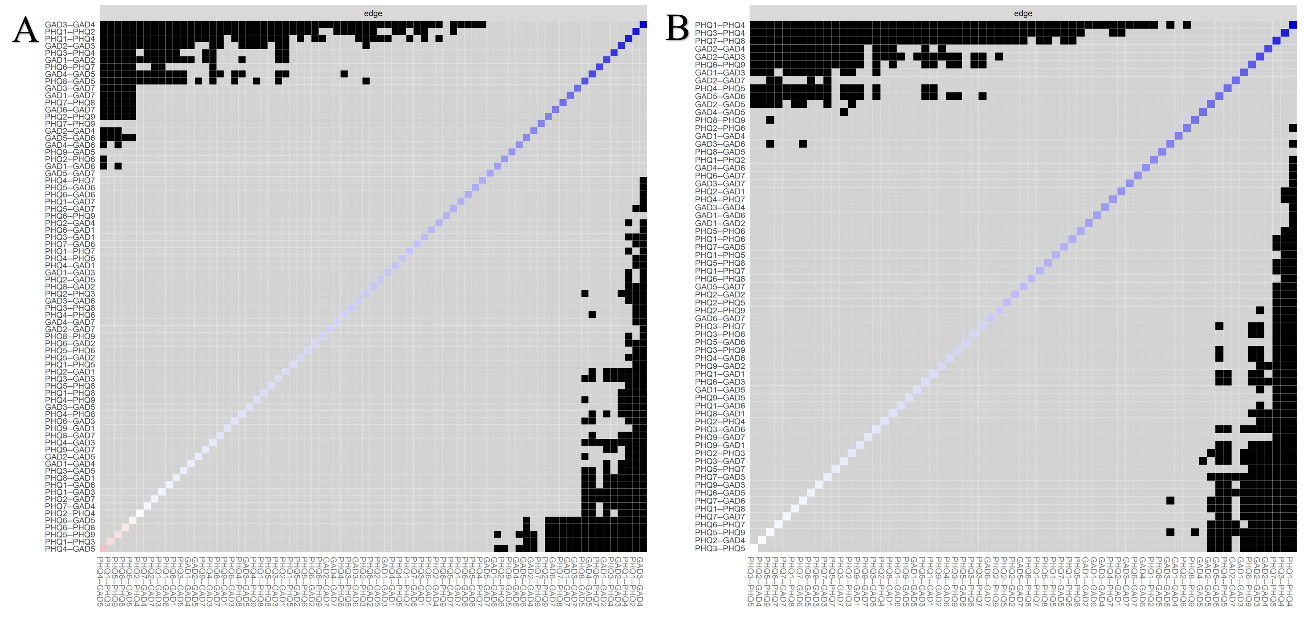
**

**Figure S1.** Nonparametric bootstrapped difference test for edges. Grey boxes indicate no significant difference, whereas black boxes indicate a statistically significant difference (*p* < 0.05). Diagonal color and saturation represent the magnitude and direction of each estimated edge. A, Female group. B, Male group.


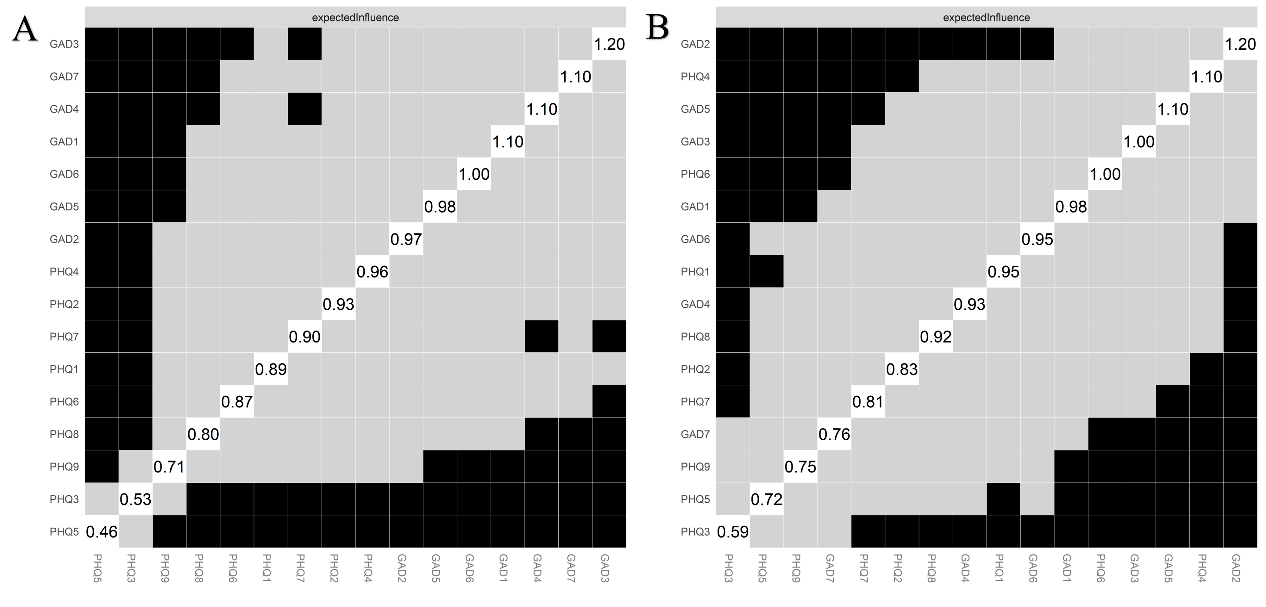


**Figure S2.** Nonparametric bootstrapped difference test for node’s centrality index (Expected influence). Grey boxes indicate no significant difference, whereas black boxes indicate a statistically significant difference (p < 0.05). Diagonal color and saturation represent the magnitude and direction of each estimated edge. A indicates all participants. A, Female group. B, Male group.


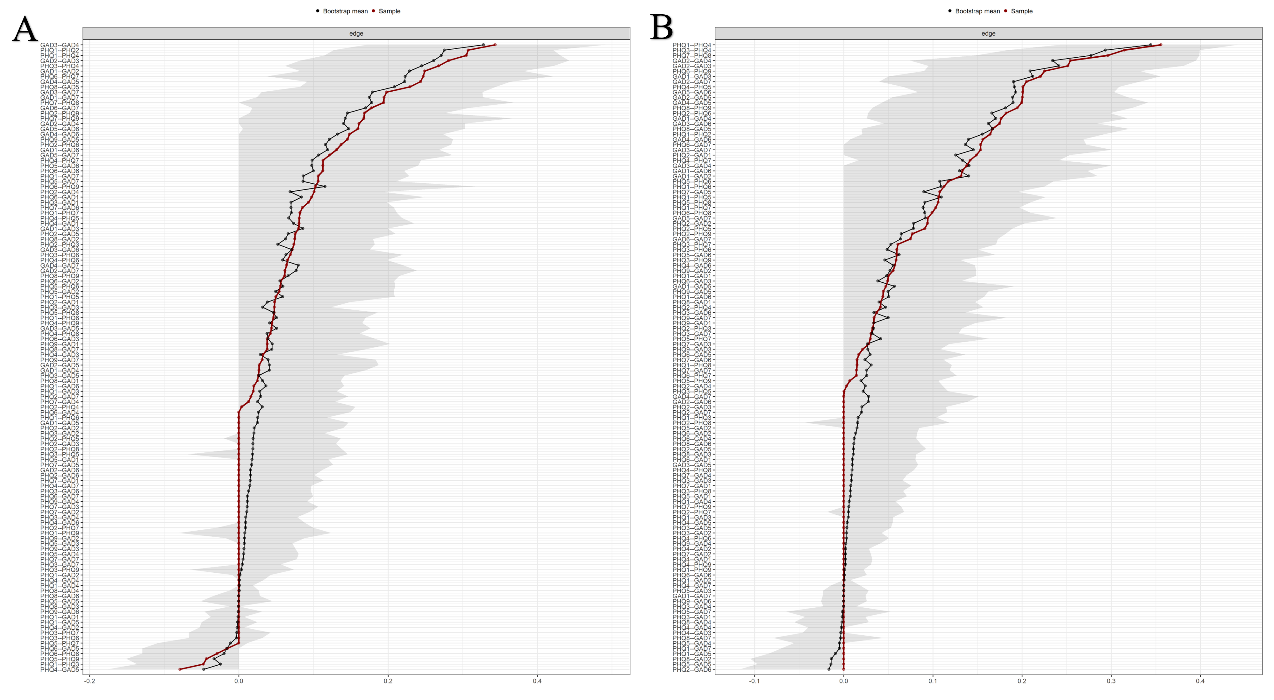


**Figure S3.** Nonparametric bootstrapped confidence intervals of estimated edges for cross-sectional networks. The red line represents the estimated edge, while the dark area indicates the 95% bootstrap confidence interval. A, Female group. B, Male group.


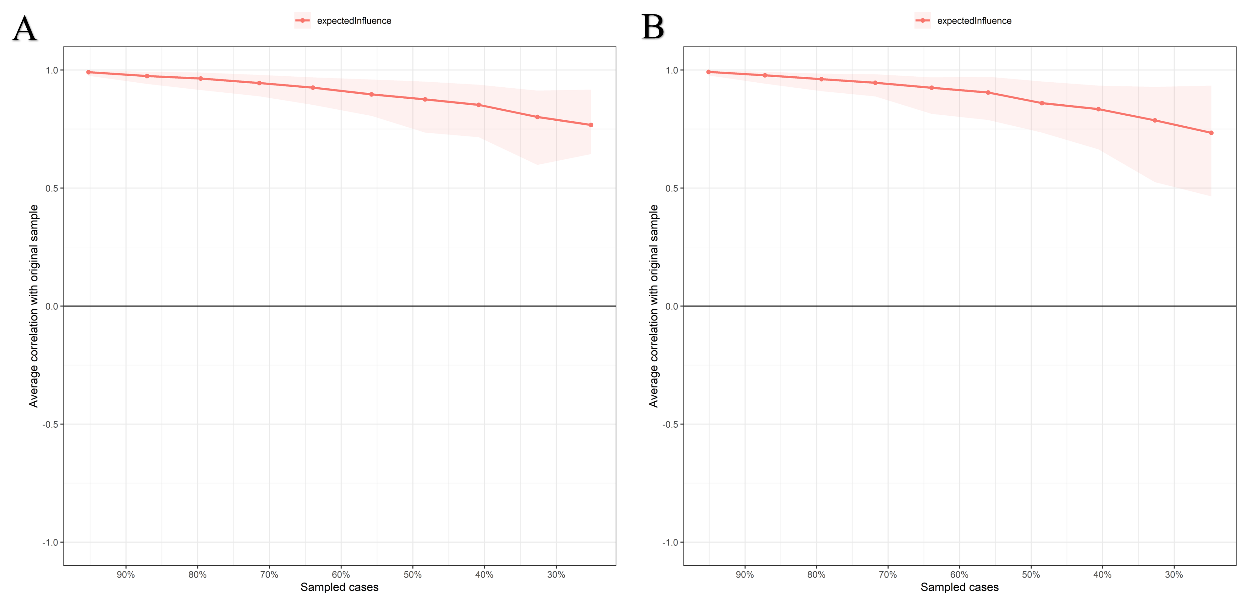


**Figure S4.** Case-dropping analysis of expected influence. The x-axis indicates the percentage of cases of the original sample included at each step. The y-axis indicates the average of correlations between the centrality indices from the original network and the centrality indices from the networks that were re-estimated after excluding increasing percentages of cases. A, Female group. B, Male group.


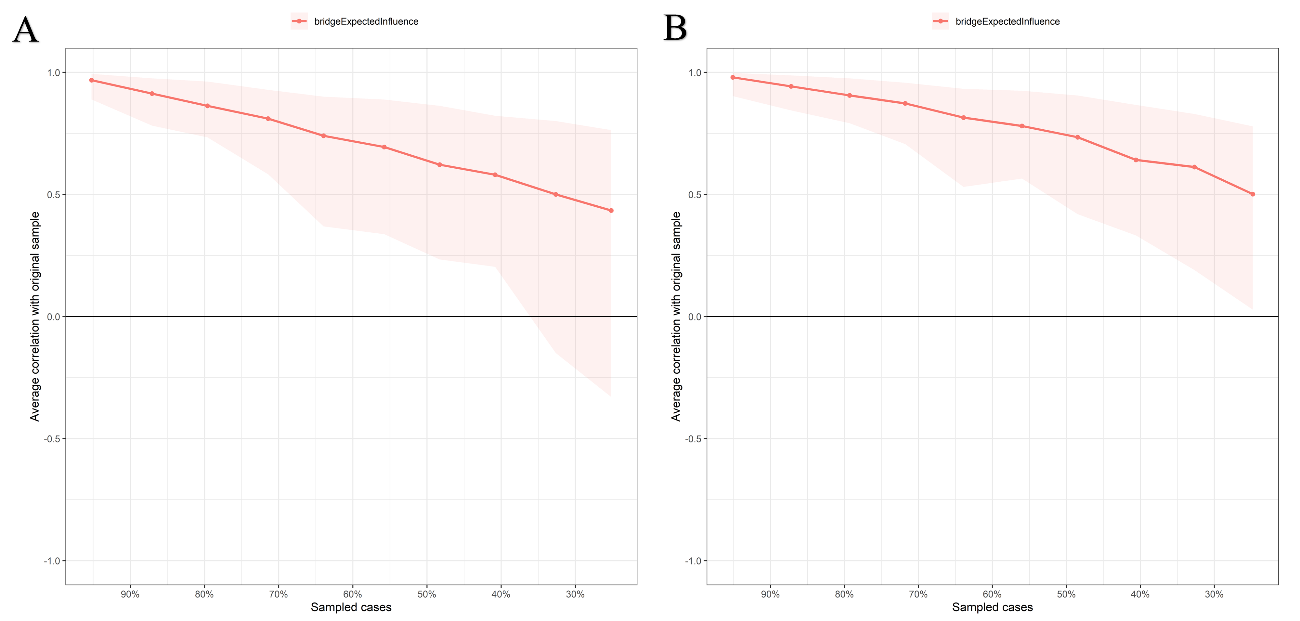


**Figure S5.** Case-dropping analysis of bridge expected influence. The x-axis indicates the percentage of cases of the original sample included at each step. The y-axis indicates the average of correlations between the centrality indices from the original network and the centrality indices from the networks that were re-estimated after excluding increasing percentages of cases. A, Female group. B, Male group.
